# Expression of Mesothelioma-Related Markers in Meningiomas: An Immunohistochemical Study

**DOI:** 10.1155/2014/968794

**Published:** 2014-04-30

**Authors:** Eman Abdelzaher, Dina Mohamed Abdallah

**Affiliations:** Department of Pathology, Faculty of Medicine, University of Alexandria, Alexandria 21533, Egypt

## Abstract

*Background*. Meningiomas are common intracranial tumors. Recently, histogenetic and phenotypic similarities between meningiomas and mesotheliomas have been proposed. We were interested in whether these similarities are reflected on the immunohistochemical level, which would add new potentially diagnostic markers for meningiomas. *Methods*. The expression of mesothelioma-related markers (D2-40, Calretinin, Keratin 5/6, WT1, and Methotheioma-Ab1) was investigated in 87 cases of meningiomas and compared to EMA expression. *Results*. 73.6% of meningioma cases were grade I, 20.7% were grade II, and 5.7% were grade III. 83.9% of meningioma cases were classical and 16.1% had special nonmeningothelial features. D2-40 was expressed in 37.9% of cases and was significantly restricted to classical meningiomas. Calretinin and WT1 were negative while Keratin 5/6 and Mesothelioma-Ab1 were weakly expressed in classical variants (5.7% and 3.4%, resp.). EMA was consistently expressed in all cases. Its expression was significantly higher than that of mesothelioma-related markers; this held true also when D2-40 expression was considered separately. *Conclusions*. Mesothelioma-related markers are not extensively expressed in meningiomas, a finding that argues against their proposed histogenetic and phenotypic similarities. Compared to EMA, the significantly lower expression of mesothelioma-related markers and their restricted expression to classical meningioma variants hamper their potential future use as diagnostic markers for meningioma.

## 1. Introduction

Meningiomas are among the most commonly diagnosed primary intracranial neoplasms [1]. Recent studies have proposed that meningiomas share common histogenetic and phenotypic characteristics with mesotheliomas [2].

Arachnoid (meningothelial) cells of the arachnoid membrane are the presumed histogenetic origin of meningiomas [3]. Despite their controversial embryogenesis, arachnoid cells differentiate to form a thin membranous structure covering the surfaces of the brain and spinal cord. In this regard, they resemble mesothelial cells that cover the surfaces of the pleural and peritoneal cavities [2].

Phenotypically, meningiomas and mesotheliomas have dual epithelial and mesenchymal differentiation which is also reflected by their immunohistochemical coexpression of epithelial and mesenchymal markers [4–6]. In addition, both tumors share, on the ultrastructural level, the presence of desmosomes and intermediate filaments [2, 5, 7].

Although conventional stains are the mainstay for pathologic diagnosis, immunohistochemistry has played a major role in improving diagnostic accuracy in general and specialized surgical pathology. The judicious use of a panel of selected immunostains is unquestionably helpful in diagnostically challenging cases [8].

The present study was performed to investigate the expression of mesothelioma-related markers in meningiomas in order to verify whether the proposed similarities between meningioma and mesothelioma are reflected on the immunohistochemical level, which would add new markers of potential diagnostic utility for meningiomas.

## 2. Materials and Methods

### 2.1. Patients and Tissue Samples

The present study comprised 87 retrospective cases of meningiomas obtained from Egyptian patients. Complete clinical, neuroimaging, and operative data were available for all patients.

Histopathological typing and grading were done according to the WHO criteria [9]. Ancillary immuno- and histochemical stains were applied whenever appropriate. Problematic cases and pure cases of special variants were excluded.

### 2.2. Immunohistochemistry

Tissue sections were deparaffinized in xylene and rehydrated through graded ethanol series. Hydrogen peroxide was applied to block endogenous peroxidase activity. Heat-induced epitope retrieval involved immersion of tissue sections in a preheated buffer solution (1 mM EDTA, pH 8.0. or 10 mM citrate buffer, pH 6.0. according to manufacturer's instructions). Immunohistochemical staining was performed using an avidin-biotinylated immunoperoxidase methodology. The primary antibodies (Thermo Scientific, USA) included EMA Ab-2 (Clone GP1.4), anti-D2-40 (Clone D2-40), Mesothelioma Ab-1 (Clone HBME-1), WT1 Ab-5 (Clone 6F-H2), Keratin 5/6 Ab-2 (Clone D5/16 B4), and Calretinin (Clone SP13). The bound antibodies were detected by the UltraVision Detection System Antipolyvalent, HRP/DAB (Ready-To-Use) (Thermo Scientific, USA). Negative and positive controls (mesothelioma for mesothelioma-related markers and breast carcinoma for EMA) were included in all runs.

### 2.3. Evaluation of Immunohistochemical Staining

The proportion of immunoreactive cells observed in each tumor was recorded as follows: (0): no immunoreactive cells, (1): 1%–25%, (2): 26%–50%, and (3): more than 50% immunoreactive cells. The staining intensity was graded using a three-tiered system as weak (1), moderate (2), and intense (3). A total score for each case was calculated by adding the two aforementioned scores. In addition, the staining pattern (cytoplasmic +/or membranous) was recorded [2].

### 2.4. Statistical Analysis

Statistical analyses were performed using SPSS Statistics 21. Quantitative data were described using median (*Mdn*), minimum, and maximum as well as mean (*M*) and standard deviation (*SD*). Qualitative data were described using number and percentage. Correlation between quantitative ordinal data was tested using Spearman's rho test. Mann-Whitney test was used to compare quantitative ordinal variables between two groups. Cochran's *Q* test was used to test for differences among the frequencies of expression of the studied six markers (pairwise comparisons were tested using McNemar's tests after Bonferroni's correction). Friedman's test was used to compare the total scores of the studied six markers (pairwise comparisons were tested using Wilcoxon-signed rank test after Bonferroni's correction). Significance test results are quoted as two-tailed probabilities and judged at *α* = 0.05.

## 3. Results 

### 3.1. Clinicopathological Findings

The age of the patients ranged from 20 to 80 years (*Mdn* = 50, *M* = 51.138, and *SD* = 11.752). About two-thirds (67.8%) of the patients were females and one third (32.2%) were males with a M : F ratio = 1 : 2.1.

All the studied cases were cranial in location. Neuroimaging and operative data showed that 80 patients (92%) had supratentorial tumors and 7 patients (8%) had infratentorial tumors.

According to the WHO grading system [9], 64 cases (73.6%) were grade I, 18 cases (20.7%) were grade II, and five cases (5.7%) were grade III. Overall, transitional meningioma was the most frequently encountered histopathological type (29 cases, 33.3%) followed by the meningothelial (19 cases, 21.8%) and fibrous (14 cases, 16.1%) types. The studied cases were divided into two broad histopathological categories, classical and special. The classical category (73 cases, 83.9%) included meningioma types with classical histopathological features while the special category (14 cases, 16.1%) included types with nonmeningothelial features such as microcystic, chordoid, and papillary types.

### 3.2. Results of Immunohistochemistry (Figure 1)

EMA was positive in all (100%) of the studied cases. Total staining score ranged from 2 to 6 (*M* = 4.345, *SD* = 0.962, and *Mdn* = 4). EMA staining pattern was cytoplasmic in 32 cases (36.8%) and cytoplasmic with membranous staining in 55 cases (63.2%). No significant difference was found regarding EMA expression in classical and special meningioma types (*U* = 434.000, *P* = 0.347).

EMA expression was significantly higher in tumors located infratentorially compared to supratentorial tumors (*U* = 203.000, *P* = 0.004). A significant negative correlation was found between EMA expression and grade (*ρ* = −0.356, *P* = 0.001).

D2-40 positively stained 33 cases (37.9%) with a staining score ranging from 0 to 5 (*M* = 1.115, *SD* = 1.543, and *Mdn* = 0). D2-40 expression was restricted to classical meningiomas in the meningothelial-transitional-fibrous spectrum and it was lacking in special meningioma types; this finding was statistically significant (*U* = 280.000, *P* = 0.002). D2-40 staining pattern was cytoplasmic in 20 cases (22.9%) and cytoplasmic with membranous staining in 13 cases (14.9%).

EMA and D2-40 had similar staining pattern in 16 cases (48.8%); however this finding was not statistically significant (observed agreement = 48.4%, Kappa = 0 .014, and *P* = 0.930).

The relation between D2-40 expression on one hand and tumor's location and grade on the other hand was statistically insignificant (*U* = 322.500, *P* = 0.141 and *ρ* = 0.016, *P* = 0.883, resp.).

Expressions of both EMA and D2-40 in meningioma cases were not significantly correlated with age (*ρ* = −0.103, *P* = 0.315 and *ρ* = −0.124, *P* = 0.225, resp.) or sex of the patients (*U* = 836.000, *P* = 0.080 and *U* = 969.000, *P* = 0.465, resp.).

As regards the remaining mesothelioma-related markers, Keratin 5/6 and Mesothelioma-Ab1 antibodies were positive in 5 (5.7%) and 3 (3.4%) cases, respectively. Among the positively stained cases, the staining pattern for both markers was predominantly cytoplasmic and total scores ranged from 2 to 3. The expression of both markers was restricted to the transitional and meningothelial types.

Calretinin and WT1 antibodies were negative in all cases.

Using Cochran's *Q* test (Table 1), there was a significant difference in the frequency of expression of the studied markers among meningioma cases (*χ* ^2^(5) = 329.7, *P* < 0.005). A pairwise comparison (at *P* = 0.05) revealed that the frequency of EMA expression in meningioma cases was significantly higher than the expression of mesothelioma-related markers.

The frequency of D2-40 expression was significantly lower than that of EMA expression on one hand and was significantly higher than the expression of the remaining four mesothelioma-related markers on the other hand.

**Figure 2:**
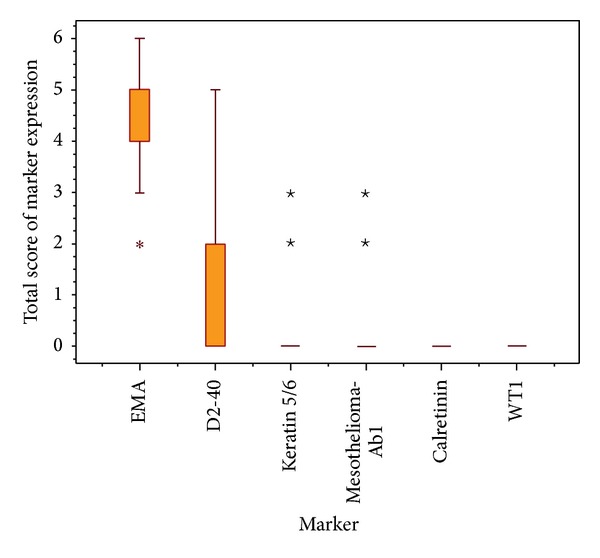
Box plot chart showing the distribution of total scores of different marker expressions in meningioma cases.

Considering the total score of different marker expressions (Figure 2), a significant difference was found in the total score distributions of the studied markers among meningioma cases (Friedman's test *χ*_5_^2^ = 361.1, *P* < 0.001). A pairwise comparison (at *P* = 0.05) revealed that the median EMA total score was significantly the highest among all other markers.

In addition, the median D2-40 total score was significantly lower than that of EMA and was significantly higher than those of the remaining four mesothelioma-related markers.

## 4. Discussion 

Meningiomas constitute approximately one-fourth of all primary intracranial tumors [10]. In the present study, most patients were adult females with predominant affection of the supratentorial region. Histologically, grade I meningiomas were the most common with the transitional subtype being the most prevalent. These findings conform to previous studies [11].

Several histogenetic and phenotypic similarities between meningiomas and mesotheliomas are recognized [2, 5].

Histogenetically, both tumors arise from cells (arachnoidal cells in meningiomas and mesothelial cells in mesotheliomas) that are capable of differentiation and proliferation to cover extensive surfaces with a thin membranous structure [2].

Phenotypically, meningiomas and mesotheliomas are neoplasms that show mesenchymal and epithelial features; in that epithelioid polygonal cells are intermingled with fibroblast-like spindle cells in varying proportions [5].

We were interested in whether these similarities are also reflected on the immunohistochemical level.

Not only the number of previous studies tackling this point is few, but also a limited panel of mesothelioma-related markers was employed [2, 12, 13].

In this study, the expression of well-established mesothelioma markers (Calretinin, Keratin 5/6, and WT1) in addition to new markers (D2-40 and Mesothelioma Ab1) [14–17] was investigated in 87 meningioma cases and compared to EMA expression.

To the best of our knowledge, this is the first study that investigates the expression of this panel of mesothelioma-related markers in meningiomas.

EMA is widely used in clinical practice to confirm the diagnosis of meningiomas. Unfortunately, EMA is not tumor-specific; therefore, new diagnostic markers for meningiomas are constantly pursued [18].

In line with previous studies [2, 19], EMA was consistently expressed in all of the studied cases with a cytoplasmic staining pattern ± membranous accentuation.

Previous reports have shown that EMA positivity in meningiomas ranged from 50% to 100% [18, 20, 21].

EMA expression was significantly higher in tumors located infratentorially compared to supratentorial tumors. Could this reflect histogenetic differences between supra- and infratentorial meningiomas? Further studies are needed to answer this question.

It has been postulated that the embryogenesis of the human meninges probably differs depending on various levels of the neuraxis. Both mesodermal cells and neural crest-derived cells are presumed to contribute in various proportions to the genesis of meningeal tissue [3].

A significant negative correlation was found between total score of EMA expression and tumor's grade. This contrasts with the study of Rivera et al. [19]. With further investigation, quantitative estimation of EMA expression may prove to be of use as a prognostic marker aiding distinction of low grade from high grade meningiomas.

EMA expression did not differ significantly in classical and special meningioma types. This could be explained by the conclusion drawn by Mennel et al. [22] who stated that meningiomas, despite of many different particular features, have a common and uniform cellular make-up demonstrated with various morphological methods.

D2-40 antibody (also known as podoplanin) is a type I integral membrane glycoprotein that is raised against an oncofetal antigen (the M2A antigen). D2-40 has been found to stain a diverse collection of normal and neoplastic tissues. In recent years, it has been utilized widely as an immunohistochemical marker for lymphatic endothelium as well as mesothelioma and germ cell tumors [13, 17, 23, 24].

In contrast to the few previous studies that investigated D2-40 expression in meningiomas [2, 12, 25], our study showed a lower (37.9%) frequency of D2-40 expression, yet with a more or less similar staining pattern.

Shintaku et al. [2] found D2-40 positivity in 91.7% of meningioma cases. Bellucci et al. [25] have reported strong and diffuse expression of D2-40 in 100% of meningioma cases. Similar results were reported by Shibahara et al. [12].

On the other hand, Mishima et al. [26] reported negative D2-40 expression in meningioma of the pineal region.

This discrepancy in D2-40 expression in different studies can be the result of different localization of the neoplasms or a difference in the monoclonal antibodies used in these studies [2].

Both EMA and D2-40 are membrane-associated proteins. The cytoplasmic reactivity demonstrated for both markers in our study was also reported by Shintaku et al. [2] who presumed that this apparently intracytoplasmic immunoreactivity is most likely to represent reactivity localized on plasma membranes showing intricate interdigitations with the neighboring cells.

Despite that EMA and D2-40 had similar staining pattern in 48.8% of the cases, this finding was not statistically significant. On the other hand, Shintaku et al. [2] reported that immunoreactivity for D2-40 corresponded well to that for EMA but was more crisp or sharply delineated and clear compared with that for EMA.

In addition, and in contrast to EMA, no significant association was found between D2-40 expression and tumor's location, further accentuating the differences between the two markers.

In our study, D2-40 expression was significantly restricted to classical meningioma types in the meningothelial-transitional-fibrous spectrum. Our results are in general agreement with the study of Shintaku et al. [2]. It is noteworthy however that their study included only classical meningioma types.

The other histopathological variants were negative for D2-40, including special meningioma types such as chordoid meningioma. A similar result was reported by Hayashi et al. [27] who found negative D2-40 expression in chordoid meningioma. On the other hand, several previous reports have demonstrated that chordoid meningiomas often express D2-40 [23, 28, 29].

In agreement with Shintaku et al. [2], our study showed that the immunoreactivity for D2-40 did not show significant changes among the various histological grades.

Calretinin is a calcium-binding protein of 29 kDa belonging to a calmodulin superfamily [30]. Although it has been widely used as an immunohistochemical marker for mesothelioma, there have been few reports on Calretinin expression in brain tumors, in particular, meningioma [2, 30].

In our study, all cases lacked Calretinin expression. Shintaku et al. [2] have reported that Calretinin was not found in most cases of meningioma, and, even in positive cases, it was restricted to the nuclei and perinuclear cytoplasm of a small number of cells.

Cytokeratins are intracytoplasmic intermediate filaments expressed in mesothelia, epithelia, and tumors derived from these tissues. Keratin 5/6 is specifically expressed in mesothelial derivatives [16].

Rarely, if ever do classical meningiomas express cytokeratins, certain subtypes as secretory meningiomas show characteristic pattern of cytokeratin expression in the inclusions and their encircling cells [9, 31, 32].

Previous studies have reported controversial results regarding the expression of cytokeratins in chordoid and papillary meningiomas [28, 29, 33–36].

Keratin 5/6 expression has not been previously studied in intracranial meningiomas. In our study, a weak expression was demonstrated in a minority of cases. Subtypes that showed reactivity were the transitional and meningothelial variants.

Fox et al. [37] have reported negative Keratin 5/6 expression in two cases of ectopic cutaneous meningiomas.

The Wilms tumor product-1 (WT1) is expressed in fetal and adult tissues such as mesothelium, spleen, and glomerular cells of the kidney. The value of WT1 as a diagnostic marker for mesothelioma has been previously confirmed [16].

In the current study, WT1 was negative in all the studied histopathological types of meningioma. The study of Singh et al. [38] is the only report in the published literature investigating the immunohistochemical expression of WT1 in fibroblastic meningiomas. The authors have shown that fibroblastic meningiomas were negative for WT1.

The negative to rare expression of well-established mesothelioma-related markers (Calretinin, Keratin 5/6, and WT1) demonstrated in meningioma cases in our study contrasts with their known patterns of expression in mesothelioma [14–16].

The mouse monoclonal antibody mesothelioma Ab-1 (HBME-1) was raised using a suspension of cells from an epithelial mesothelioma as an immunogen. The antibody recognizes an undetermined antigen abundant on the surface of normal and neoplastic mesothelial cells which is also present in other epithelial cells [39, 40]. This antibody has been used in the differential diagnosis of mesothelioma cases [16]. No previous studies have investigated its expression in meningiomas.

In our study, Mesothelioma Ab-1 was focally and weakly expressed in few cases. The positive cases were restricted to transitional and meningothelial subtypes. This contrasts with its strong expression in mesothelioma cases [39]. Also the pattern of cytoplasmic expression demonstrated in our study is different from the characteristic “thick brush border” pattern seen in mesotheliomas which correlates with the presence of long and abundant microvilli on mesothelioma cells [39].

In the present study, the frequency and total score of EMA expression in meningioma cases were significantly higher than those of the expression of mesothelioma-related markers. This holds true also when D2-40 expression was considered separately.

From the above, we can conclude that the expression of mesothelioma-related markers in meningiomas is neither extensive nor comparable to EMA expression in meningiomas or to their known patterns of expression in mesotheliomas. This argues against the proposed histogenetic and phenotypic similarities between meningiomas and mesotheliomas.

The present study was also aiming at verifying the potential diagnostic role of mesothelioma-related markers in meningiomas.

Although D2-40 was expressed in a subset of classical meningiomas and its expression was significantly higher than that of the remaining four mesothelioma-related markers, nevertheless, it cannot be relied on as a diagnostic marker for meningioma.

Its relative low frequency and restricted expression to classical meningioma subtypes limit its potential diagnostic usefulness.

Diagnosis of meningiomas with classic features is usually straightforward with little need of immunohistochemical confirmation. On the other hand, the important role for immunohistochemistry in meningioma variants dominated by unusual features is obvious [41].

In addition, other mesothelioma-related markers studied were either negative or weakly and rarely expressed in classical meningiomas.

Taken together, our study suggests that mesothelioma-related markers cannot be relied on as diagnostic markers for meningiomas, and EMA, although not specific, is consistently and significantly expressed in meningiomas and thus remains superior in its diagnostic value. This understandably emphasizes that additional markers of meningothelial differentiation are sorely needed.

## Figures and Tables

**Figure 1:**
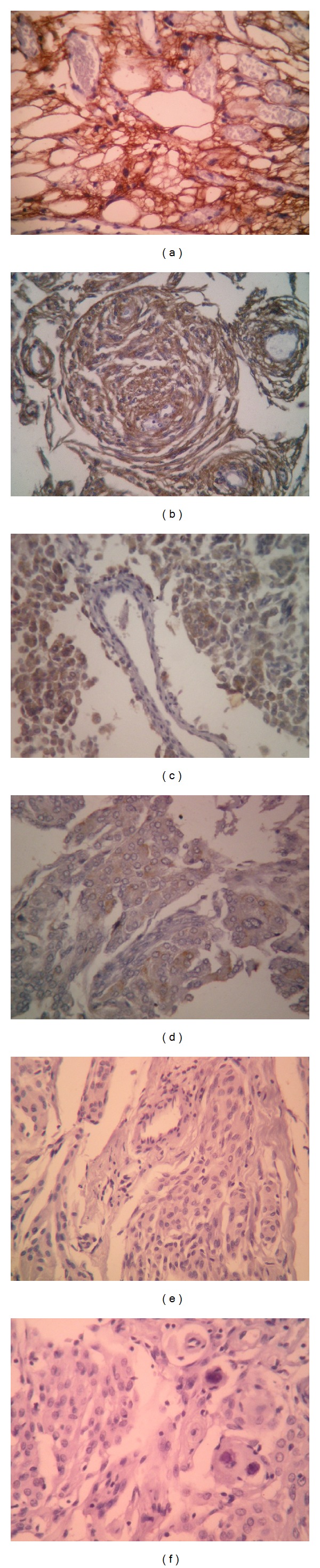
Immunohistochemical expression of EMA and mesothelioma-related markers in meningioma cases: (a) microcystic meningioma showing positive cytoplasmic and membranous staining for EMA (×200), (b) transitional meningioma featuring cytoplasmic reactivity for D2-40 (×200), ((c) and (d)) meningothelial cells showing focal cytoplasmic staining for Keratin 5/6 and Mesothelioma-Ab1, respectively (×100 and ×200), and ((e) and (f)) negative staining for Calretinin and WT1, respectively (×200).

**Table 1:** Comparison between the frequencies of marker expression in meningioma cases using Cochran's *Q* test.

	Marker expression		Proportion (%)	Pair-wise comparisons among different groups at *P* = 0.05
	−ve	+ve		
(1) EMA	0	87	100.00	(2) (3) (4) (5) (6)
(2) D2-40	54	33	37.93	(1) (3) (4) (5) (6)
(3) Keratin 5/6	82	5	5.75	(1) (2)
(4) Mesothelioma-Ab1	84	3	3.45	(1) (2)
(5) WT1	87	0	0.00	(1) (2)
(6) Calretinin	87	0	0.00	(1) (2)

Minimum required difference (%): 20.2802.
